# Correction: The Rhizosphere Selects for Particular Groups of *Acidobacteria* and *Verrucomicrobia*

**DOI:** 10.1371/journal.pone.0094514

**Published:** 2014-04-01

**Authors:** The PLOS ONE Staff

**Notice of Republication**

This article was republished on March 7th, 2014, due to the figures missing in the PDF version of the previously published article. Please download the PDF again to view the correct figures. The originally published, uncorrected article and the republished, corrected article are provided here for reference.

## Supporting Information

File S1 Originally published, uncorrected article
(PDF)

File S2 Republished, corrected article
(PDF)
