# Supplementary material for: Correction: The Rhizosphere Selects for Particular Groups of Acidobacteria and Verrucomicrobia
Source: PLoS One. 2014 Apr 1;9(4):e94514. doi: 10.1371/journal.pone.0094514 (PMC3972092; doi:10.1371/journal.pone.0094514)
Supplement: File S1 — Originally published, uncorrected article (PDF) [file pone.0094514.s001.pdf]

The Rhizosphere Selects for Particular Groups of *Acidobacteria* and *Verrucomicrobia*

Ulisses Nunes da Rocha[1,2☯], Caroline M. Plugge[3], Isabelle George[4], Jan Dirk van Elsas[5], Leonard Simon van Overbeek[1*☯]

1 Plant Research International, Wageningen University and Research Centre, Wageningen, The Netherlands, **2** Lawrence Berkeley National Laboratory, Center for Environmental Biotechnology, Berkeley, California, United States of America, **3** Laboratory for Microbiology, Wageningen University and Research Centre, Wageningen, The Netherlands, **4** Laboratoire d'Ecologie des Systèmes Aquatiques, Université Libre de Bruxelles, Bruxelles, Belgium, **5** Department of Microbial Ecology, Centre for Ecological and Evolutionary Studies, Groningen University, Groningen, The Netherlands

Abstract

There is a lack in our current understanding on the putative interactions of species of the phyla of *Acidobacteria* and *Verrucomicrobia* with plants. Moreover, progress in this area is seriously hampered by the recalcitrance of members of these phyla to grow as pure cultures. The purpose of this study was to investigate whether particular members of *Acidobacteria* and *Verrucomicrobia* are avid colonizers of the rhizosphere. Based on previous work, rhizosphere competence was demonstrated for the *Verrucomicrobia* subdivision 1 groups of *Luteolibacter* and *Candidatus* genus *Rhizospheria* and it was hypothesized that the rhizosphere is a common habitat for *Acidobacteria* subdivision 8 (class *Holophagae*). We assessed the population densities of *Bacteria*, *Verrucomicrobia* subdivision 1 groups *Luteolibacter* and *Candidatus* genus *Rhizospheria* and *Acidobacteria* subdivisions 1, 3, 4, 6 and *Holophagae* in bulk soil and in the rhizospheres of grass, potato and leek in the same field at different points in time using real-time quantitative PCR. Primers of all seven verrucomicrobial, acidobacterial and holophagal PCR systems were based on 16S rRNA gene sequences of cultivable representatives of the different groups. *Luteolibacter*, *Candidatus* genus *Rhizospheria*, subdivision 6 acidobacteria and Holophaga showed preferences for one or more rhizospheres. In particular, the Holophaga 16S rRNA gene number were more abundant in the leek rhizosphere than in bulk soil and the rhizospheres of grass and potato. Attraction to, and colonization of, leek roots by *Holophagae* strain CHC25 was further shown in an experimental microcosm set-up. In the light of this remarkable capacity, we propose to coin strain CHC25 *Candidatus* Porrumbacterium *oxyphilus* (class *Holophagae*, Phylum *Acidobacteria*), the first cultured representative with rhizosphere competence.

Citation: Nunes da Rocha U, Plugge CM, George I, van Elsas JD, van Overbeek LS (2013) The Rhizosphere Selects for Particular Groups of *Acidobacteria* and *Verrucomicrobia*. PLoS ONE 8(12): e82443. doi:10.1371/journal.pone.0082443

Editor: Paul Jaak Janssen, Belgian Nuclear Research Centre SCK/CEN, Belgium

Received June 6, 2013; **Accepted** October 25, 2013; **Published** December 13, 2013

Copyright: © 2013 Nunes da Rocha et al. This is an open-access article distributed under the terms of the Creative Commons Attribution License, which permits unrestricted use, distribution, and reproduction in any medium, provided the original author and source are credited.

Funding: This research was conducted within the Netherlands Genomic Initiative(NGI)-financed program of 'Ecogenomics', and the Research Program on Sustainable Agriculture (KB4), financed by the Dutch Ministry of Agriculture, Nature and Food Quality. The funders had no role in study design, data collection and analysis, decision to publish, or preparation of the manuscript.

Competing interests: The authors have declared that no competing interests exist.

* E-mail: leo.vanoverbeek@wur.nl

☯ These authors contributed equally to this work.

Introduction

The phyla *Acidobacteria* and *Verrucomicrobia* are among the most dominant bacterial groups present in most soils [1-6]. The phylum *Acidobacteria* consists of at least 26 monophyletic groups, so called subdivisions, whereas the *Verrucomicrobia* have at least seven subdivisions [5,7]. Both phyla are intriguing prokaryotes given their presumed roles in soil ecosystems and also because the vast majority of species belonging to these phyla remains uncultured to date [4,8]. In particular, very little is known about the ecological roles of members of the *Acidobacteria* and *Verrucomicrobia* in plant-soil ecosystems.

Most of the available data on the ecology of *Acidobacteria* and *Verrucomicrobia* in plant-soil ecosystems comes from studies in which cultivation-independent (metagenomic) approaches were applied. For instance, members of the *Verrucomicrobia* have been shown to be present in varying plant-soil ecosystems [9-14]. Also, representatives of the phylum *Acidobacteria* were found in these systems, although they tended to be more associated with bulk, than with rhizosphere soils [13,15]. In soil, pH seemed to play an important role as a determinant of acidobacterial assemblages [5,16-20]. In addition, mineral composition [21,22], temperature [18] and nutrient availability [15,23-25] were important. The relative abundances of *Acidobacteria* in clone libraries from

pasture soil were found to be between 7 - 14% [1] and of *Verrucomicrobia* around 1.9 % [26]. In Brazilian Atlantic forest soil, such relative numbers were higher, i.e. 29 - 54% for *Acidobacteria* and 0.6 - 14% for *Verrucomicrobia* [27]. Using barcoded pyrosequencing on soil-extracted DNA, *Verrucomicrobia* operational taxonomic units (OTUs) comprised 35% of all bacterial OTUs retrieved from 112 undisturbed mineral soils sampled across different continents (North and South America, Europe and Antarctica) [28]. In that study, *Spartobacteria* and subdivision 3 were found to be the most abundant subdivisions among *Verrucomicrobia*. In 88 different soils, *Acidobacteria* contributed to 35% of total bacterial OTUs, whereas for *Verrucomicrobia* it was 0.9% [5]. In that study, *Acidobacteria* subdivision 1 accounted for 7.4% of all bacterial and for 17.6% of all acidobacterial OTUs. Considering all data, there appears to be wide variation in the contribution of both phyla and their separate subdivisions to the bacterial communities present in soil environments.

A major task is the cultivation of some of the diversity of the *Acidobacteria* and *Verrucomicrobia* detected by direct molecular approaches in soils. Thus, based on cultured representatives from soil, subdivisions 1, 2, 3, 4 and 6 of *Acidobacteria* have been found in pasture soil [4,16,29-31], and subdivision 8 (class of *Holophagae*) in the leek rhizosphere [32]. *Verrucomicrobia* subdivision 2 (*Spartobacteria*) and subdivision 3 strains were isolated from pasture soils [4,33], whereas *Verrucomicrobia* subdivision 1 strains were found in the rhizospheres of potato and leek [12,32,34]. There appears to be a distinction in verrucomicrobial and acidobacterial compositions between bulk and rhizosphere soils, as *Verrucomicrobia* subdivision 1 and *Holophagae* isolates were found in close proximity to plant roots. Rhizosphere competence was demonstrated for two distinct *Verrucomicrobia* subdivision 1 representatives, provisionally denominated *Luteolibacter* and *Candidatus* genus *Rhizospheria* [12]. Rhizosphere competence has never been demonstrated for representatives of the phylum *Acidobacteria*. It was, therefore, a challenge to explore the distribution of *Holophagae*, in relation with other groups of *Acidobacteria* belonging to subdivisions 1, 3, 4, 6 and of *Verrucomicrobia* subdivision 1 *Luteolibacter* and *Candidatus* genus *Rhizospheria*, in the rhizosphere of different plants. For that purpose, we used real time quantitative PCR (qPCR), accurately calibrated by making use of culturable representatives of these groups [12,32].

In this study, we addressed the question whether the cultured *Holophagae* strains are rhizosphere-competent, in analogy to the study performed on two different *Verrucomicrobia* subdivision 1 groups [12]. We define rhizosphere competence as the ability of bacteria to move towards plant roots and to grow on root-released nutrients [35]. The study includes data from the field, focusing on different *Acidobacteria* subdivisions and *Verrucomicrobia* subdivision 1, and from a rhizosphere microcosm study. The latter was set up to further explore the possible competence in the leek rhizosphere of a soil-introduced *Holophagae* strain.

Materials and Methods

Field site, soil and plant sampling, sample processing and analysis procedures

The field site was located at the experimental farm 'De Droevendaal' (51°59'32"N, 5°40'12''E), Wageningen, The Netherlands. The soil was a loamy sand containing 2% organic matter, with a water holding capacity of 25% and a pH (KCl) of 4.8. Before onset of the experiment, the field (21 by 25 m) was covered with a permanent grass ley (commercial mix, containing *Lolium perenne* as the main plant species) and maintained under agricultural management practices. Then the field was divided into 16 plots of 4 by 5 m in size with a distance of 1 m between subplots and the margins of the field. Four treatments, i.e. fallow, grass, potato and leek, were in fourfold applied over the field according to a randomized scheme. Therefore, grass was removed from 12 plots, whereas it was maintained on four plots (grass). Two fallow plots were immediately planted with potato (*Solanum tuberosum* L. cultivar Agria) or leek (*Allium porrum* cultivar Kenton, Nunhems Seeds BV, The Netherlands) and the other four plots were kept fallow (non-rooted bulk soil).

Seed potatoes and leek nursery plants were planted in May 2009. Organic agricultural management practices were continued for all plots, which specifically comprehends no use of pesticides or chemical fertilizers and weekly removal of weed plants by hand. Samples from each subplot were taken in June, July and September. Samples from the potato and leek plots were taken as individual plants, whereas those from the grass and fallow soil (one per plot) were taken with a soil bore (diameter size of 7 cm) to a maximal depth of 15 cm in fallow soil. All samples were directly processed in the laboratory, where soil adhering to the grass, leek and potato roots after manual shaking of the plants was considered as rhizosphere soils. For (non-rooted) bulk soil, samples from the 5-10 cm horizons of the fallow plots were singled out. Soil pH was measured in all bulk and rhizosphere soils in 0.01 M $CaCl_2$ (1: 10 w/v ratio) according to the procedure described in [36].

Leek Rhizospere colonization by soil-indigenous *Holophagae* cells and strain CHC25 in a plant-soil microcosm

The behavior of *Holophagae* species and their representative strain, CHC25 [32], was studied near leek roots in non-sterile and sterilized Vredepeel soil with or without leek plants, using the same microcosm set up (Kuchenbuch-style) as previously described for *Luteolibacter* and *Candidatus* genus *Rhizospheria* strains in Nunes da Rocha et al. [12]. In short, non-sterile and non-inoculated soil (set up A), or sterilized soil with approximately 10^5 strain CHC25 cells per g dry soil (set up B), or with a 1 cm non-inoculated and sterilized soil layer placed between strain CHC25-inoculated soil and the membrane separating leek roots from soil (set up C), or the same as set up C, but then without leek plants (set up D). After 35 days, rings (in triplicate) were destructively sampled and soils at 0-2 mm and 10-12 mm from the nylon membrane with roots were singled out and homogenized. One-gram

subsamples were drawn for later DNA extraction and *Holophagae*-specific real-time qPCR analysis [32].

DNA extraction from soils and real-time quantitative PCR analyses

DNA from all bulk and rhizosphere soils (Vredepeel and Droevendaal soils) was extracted using the PowerSoil Isolation Kit (MO BIO Laboratories, Inc., CA, USA) following the instructions provided by the manufacturer. Quantitative PCR primers Eub338 [37] and Eub518 [38] were used for quantification of bacteria, representing ‘total bacteria’ within the domain of *Bacteria* (Table 1). Primer combinations VS1Af/ VS1Ar, VS1Bf /VS1Br and Acg8f/ Acg8r were, respectively, used for quantification of *Verrucomicrobia* subdivision 1 groups of *Luteolibacter* and *Candidatus* genus *Rhizospheria*, and of Holophaga (representing the class *Holophagae*) [12,32]. Four new primer systems, used for quantitative detection of subdivisions 1, 3, 4 and 6 acidobacteria (Table 1), were designed based on almost entire (> 1300 bp) 16S rRNA gene sequences of cultured strains IGEO12 (subdivision 1, accession number GU187028), IGEO15 (subdivision 3, GU187034), IGEO17 (subdivision 4, GU187032) and IGEO01 (subdivision 6, GU187036) (all strains are described in George et al. [29]), according to the procedure described in Nunes et al. [32]. In short, primers were validated in three steps. The first step comprehended *in silico* validation of primers. Therefore, alignments were made for each acidobacterial subdivision using 16S rRNA gene sequences of these strains and those of related bacterial groups retrieved from the SILVA database, release 102 [39]. Primers, specific for each subdivision, were designed based on conserved sequences and checked for absence on possible occurrences of mispriming events using Primer-BLAST software (http://www.ncbi.nlm.nih.gov/tools/primer-blast/). The second step comprehended validation by PCR on DNA extracts from pure culture strains. Therefore, specificity of designed primers, per subdivision, was checked with DNA from corresponding (target) and non-corresponding (non-target) strains by standard PCR amplification (Table 1). As non-target strains, all non-corresponding *Acidobacteria* and *Verrucomicrobia* subdivision 1 strains were chosen, supplemented with *Agrobacterium tumefaciens* UBAPF2 (*Alphaproteobacteria*), *Burkholderia cepacia* LMG 1222T (*Betaproteobacteria*), *Escherichia coli* E1 (*Gammaproteobacteria*), *Streptomyces griseus* IPO 857 (*Actinobacteria*), *Flavobacterium columnar* 2003/035 (*Bacteroidetes*) and *Bacillus subtilis* Bs4 (*Firmicutes*). All these strains were derived from the strain collection of Plant Research International (Wageningen, The Netherlands). The third step comprehended specificity checks on amplicon sequences derived by standard PCR with these primers from Droevendaal soil DNA extracts. Therefore, soil extracted DNA was PCR amplified and individual amplicons were cloned into the pGEM-T easy vector (Promega, WI, USA) for sequencing. A total of 192 sequences (48 per primer system) from randomly selected clones were aligned using MEGA 4 software [40] and individually compared by BlastN-assisted database searches (http://blast.ncbi.nlm.nih.gov/Blast.cgi). Finally, standard curves for each of the four primer systems were made by qPCR, based on ranges of between 10 and 10^9 cells per corresponding strain, whereas for the bacterial primer system, cells of *Pseudomonas fluorescens* Pf5 were used. Calibration curves were made in triplicate by plotting measured threshold cycle (Ct) values against ^{10}log cell number for each qPCR system. Line slopes and intercepts were calculated by linear regression analysis (Genstat 15th edition, Hemel Hempstaed, UK) and the amplification efficiency (Ae) of the different primer systems was calculated using the formula Ae = $10^{(-1/slope)}$. Theoretical dynamic ranges for all qPCR systems were determined according to Nunes da Rocha et al. [32].

Quantitative PCR systems (Table 1) were applied for molecular quantification of different *Acidobacteria* and *Verrucomicrobia* subdivisions in Droevendaal (with all eight primer systems) and Vredepeel (with only the one of *Holophagae*) soils. Therefore, DNA extracts were 10-fold diluted to approximately 5 ng per 25 µL reaction mixture, prior to running under the conditions previously described in Nunes da Rocha et al. [32]. A total of three qPCRs per primer system were run for each sample and obtained Ct values were averaged prior to conversion to log cell equivalent numbers using the appropriate regression equation for each primer system.

Statistical comparisons and multivariate analyses

Statistical comparisons, based on ^{10}log-transformed bacteria, subdivisions 1, 3, 4, 6 acidobacteria and Holophaga, *Luteolibacter* and *Candidatus* genus *Rhizospheria* cell equivalent (Ceq) numbers (expressed per g dry soil) were made between (1) different bulk soils over time, (2) between rhizospheres and bulk soils for calculation of Δ Ceq $_{rs,\,b}$ values, and (3) between fractions of total bacteria (individual population size as fraction of total bacteria within the same sample) in rhizopheres and bulk soil over time. Comparisons between rhizosphere and bulk soils sampled over time were also made on the basis of pH values. All comparisons were based on four replicate samples per treatment (rhizosphere type or bulk soil sampled over three time points).

The effects of grass, potato and leek roots in soil on the eight different populations, expressed as Δ Ceq $_{rs,\,b}$, were calculated for each population by subtraction of the log Ceq number (per g dry soil) in bulk soil from each of the corresponding rhizosphere soils. Values were presented as ‘positive’ when Ceq numbers were significantly higher in rhizosphere than in bulk soil, ‘negative’ when significantly lower, and ‘zero’ when statistically indistinguishable.

In experimentation with the plant-soil microcosms, comparisons in log-transformed Holophaga cell equivalent numbers (per g dry soil) were made between: (1) 0-2 and 10-12 mm layers of set up A, (2) 0-2 and 10-12 mm layers of set up B, (3) 0-2 and 10-12 mm layers of set up C, (4) 0-2 mm layers of set ups C and D. Averages per soil layer were based on triplicate values for each of the four microcosm set ups. Significance of differences were calculated with two-way ANOVA (Genstat 15th edition). Least significant differences were calculated from standard errors of difference. All differences were considered to be significant at levels of $P \leq 0.05$.

Table 1. Description and characteristics of the group-specific real-time (q)PCR primers 'total bacteria' targeting the domain *Bacteria*, '*Luteolibacter*' and '*Candidatus* genus *Rhizospheria*', targeting *Verrucomicrobium subdivision 1 groups* of *Luteolibacter* and *Candidatus* genus *Rhizospheria*, 'subdivisions 1, 3, 4, 6 acidobacteria and Holophaga' representing, respectively, *Acidobacteria* subdivisions 1, 3, 4, 6 and *Holophagae*.

Taxa	Target group	Sense	Primer sequence (5' – 3')	Primer name	Tm (°C)[a]	Amplicon length (bp[b])	Ae[c] (%)	Dr[d]	Reference
Bacteria	total bacteria	Forward	ACTCCTACGGGAGGCAGCAG	Eub338	57.6	200	1.91	4.17 to 9.17	[37]
		Reverse	ATTACCGCGGCTGCTGG	Eub518	54.4				[38]
Verrocumicrobia subdivision 1	*Luteolibacter*	Forward	CAGCTCGTGTCGTGAGATGT	VS1Af	60.0	199	1.98	2.26 to 8.26	[32]
		Reverse	TCTCGGTTCTCATTGTGCTG	VS1Ar	60.0				
	Candidatus genus *Rhizospheria*	Forward	GCCCGACAGGGTTGATAGTA	VS1Bf	60.0	83	1.95	2.45 to 8.45	[32]
		Reverse	CGCTTGGGACCTTCGTATTA	VS1Br	60.1				
Acidobacteria	subdivision 1	Forward	CAGGTACCCAATCCTGTCGT	Acg1f	59.8	83	98	4.21 to 9.21	This study
		Reverse	CCTTTGAGTTTCAGCCTTGC	Acg1r	60.0				
	subdivision 3	Forward	TAGGCGGTTGGGTAAGTTTG	Acg3f	60.0	100	96	4.28 to 7.28	This study
		Reverse	AGGAATTCCGCTTTCCTCTC	Acg3r	59.8				
	subdivision 4	Forward	GCACGGGTGAGTAACACGTAA	Acg4f	61.0	86	96	3.74 to 8.74	This study
		Reverse	CGCTGCATTATGCGGTATTA	Acg4r	59.7				
	subdivision 6	Forward	GAGGTAATGGCTCACCAAGG	Acg6f	59.6	193	96	4.42 to 8.42	This study
		Reverse	GTCCCGTTCGACAGGAGTT	Acg6r	60.1				
	Holophagae	Forward	TGGGATGTTGATGGTGAAAC	Acg8f	59.2	470	2.01	2.54 to 7.54	[32]
		Reverse	AGTCTCGGATGCAGTTCCTG	Acg8r	60.4				

[a] Tm, melting temperature

[b] bp, base pairs.

[c] Ae, amplification efficiency. The efficiency of the reaction was calculated by the following equation: $Ae = 10^{(-1/slope)}$; where 'slope' is the slope of the standard curve.

[d] Theoretical dynamic range (log ceq per ml) - the range of initial template concentrations over which reliable Ct values were obtained.

doi: 10.1371/journal.pone.0082443.t001

Multivariate analysis (CANOCO for Windows version 4.5, Biometris, Plant Research International, The Netherlands) was performed on all soil samples using sample type (rooted versus non-rooted soils), period of sampling, plant species (all nominal variables) and pH (numerical variable) as the 'environmental' variables and log Ceq numbers for each group (per g dry soil), as 'species' variables. Gradient lengths were calculated by detrended correspondence analysis (DCA) in a first step, and correlations between 'environmental' and 'species' variables in a second step by redundancy analysis (RDA). Monte Carlo permutation test (499 permutations) was included to calculate significance of effects on species variables.

Results

Specificity of *Acidobacteria* subdivisions 1, 3, 4 and 6 quantitative PCR primer systems

Four, of eight, qPCR primer systems (Table 1) were newly designed for the purpose of this study. From Primer-Blast analysis it was predicted that all primers targeting subdivisions 1, 3, 4 and 6 of *Acidobacteria* would specifically amplify 16S rRNA gene sequences of the targeted subdivisions. Standard PCR amplifications using the four primer systems on genomic DNA extracts from respective target strains invariably resulted in single amplicons of the expected sizes (Table 1) in the absence of any visible primer diming or other products resulting from primer mismatching (data not shown). Standard PCR amplifications with these four primer systems on genomic DNA extracts from non-target strains from different bacterial phyla (*Firmicutes, Proteobacteria*, *Bacteriodetes*) as well as from non-corresponding *Acidobacteria* and/or *Verrucomicrobia* subdivision 1 strains resulted in absence of any bands under the applied amplification conditions. Sequence comparisons of 192 amplicons, made with the four primer systems in standard PCRs with Droevendaal soil DNA extract as template, consistently revealed similarities of 96% and over with database sequences belonging to the expected subdivisions, with the exception of the primer set designed for detection of subdivision 6 species that revealed matches with the corresponding subdivision at 46 occasions, whereas at the other two occasions, sequences showed closest matches with subdivision 10 species. In total 22 distinguishable sequence groups (five of subdivision 1, seven of subdivision 3, four of subdivision 4 and six of subdivision 6), containing one to up to 18 identical sequences per group, were deposited in the EMBL Nucleotide Sequence Database and available under accession numbers FN994868 to FN994889.

Quantitative PCR on a density range of 10 – 10^9 cells per target strain for each qPCR system resulted in linear regression equations with R^2 values of 0.9841 and higher. Calculated amplification efficiency values (in %) ranged

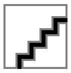

Figure 1. Dynamics of total bacteria, *Luteolibacter* and *Candidatus* genus *Rhizospheria* and of subdivisions 1, 3, 4, 6 acidobacteria and holophaga in fallow field soil over time measured by qPCR. Bact, total bacteria; Lut, *Luteolibacter*; Rhiz, ***Candidatus*** genus *Rhizospheria*; AS1, AS3, AS4, AS6, respectively, subdivision 1, 3, 4, 6 acidobacteria; Holo, holophaga. Bars on top of columns represent standard errors of means; *, significant decrease and **, significant increase in comparison with respective samples drawn in May.

doi: 10.1371/journal.pone.0082443.g001

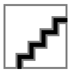

Figure 2. Effects of grass, potato and leek rhizospheres on abundances of total bacteria, *Luteolibacter* and *Candidatus* genus *Rhizospheria* and of subdivisions 1, 3, 4, 6 acidobacteria and holophaga (expressed as Δ Ceq $_{rs, b}$ values, i.e. log cell equivalent numbers from bulk soil subtracted from those from corresponding rhizospheres). G, P, L: Respectively, grass, potato, leek; 0: Δ Ceq rs, b value is zero.
Bact, total bacteria; Lut, *Luteolibacter*; Rhiz, *Candidatus* genus *Rhizospheria*; AS1, AS3, AS4, AS6, respectively, subdivision 1, 3, 4, 6 acidobacteria; Holo, holophaga.

doi: 10.1371/journal.pone.0082443.g002

between 1.91 and 2.01 and lowest and highest values in calculated dynamic ranges (in log cell equivalents per ml) were between, respectively, 2.26 to 9.21 (Table 1).

Plant growth and soil pH in the experimental field plot

Potato and leek plants grew normally in the field plots during the experimental period from May - September 2009 in the absence of any visible harm caused by pests, pathogens or abiotic stressors. The average pH values over the different samples revealed significant effects of plant released protons in the rhizosphere (related to plant growth), but no effect of time. The average pH values were significantly (n=4, P≤ 0.05) lower in bulk soil (4.77 ± 0.09) and in the rhizospheres of potato (4.78 ± 0.33) and leek (4.65 ± 0.19) than in that of grass (5.07 ± 0.16).

Dynamics of *Bacteria*, *Acidobacteria* subdivision 1, 3, 4, 6 and *Holophagae*, *Luteolibacter* and *Candidatus* genus *Rhizopheria* groups in bulk soil

The qPCR analyses (data expressed in log cell equivalents, Ceq, per g dry soil), revealed that total bacterial numbers significantly (n=4, P≤ 0.05) declined over time from 9.70 (May) to 8.61 (September) (Figure 1). *Luteolibacter* and *Candidatus* genus *Rhizospheria* numbers also declined over time, respectively, from 5.67 and 5.92 in May to 3.16 and 5.02 in September. The dynamics of the acidobacterial subdivisions 1, 3, 4, 6 and Holophaga was diverse. Subdivisions 3, 4 and 6 declined over time, respectively, from 8.37, 8.62 and 9.55 in May to 7.80, 6.74 and 7.78 in September. This in contrast to subdivision 1 acidobacteria, of which the numbers roughly remained the same (between 8.32 in May to 8.57 in September) and to Holophaga, whose numbers after an initial decrease, significantly increased, from 5.17 in May to 5.42 in September. Removal of the grass layer from the fallow plot thus led to a unique increase in estimated *Holophagae* cell numbers.

Effect of different Rhizospheres on *Bacteria*, *Acidobacteria* subdivision 1, 3, 4, 6, *Holophagae*, *Luteolibacter* and *Candidatus* genus *Rhizospheria* estimated cell numbers

Positive Δ Ceq $_{rs, b}$ values (log cell equivalent numbers from bulk soil subtracted from those from corresponding rhizospheres) were found for bacteria across all three plant species and sampling periods, indicating that plant roots stimulated bacterial growth in soil (Figure 2). For *Luteolibacter*, positive Δ Ceq $_{rs, b}$ values were also found at all occasions, i.e. in the rhizospheres of all three plant species in June, July and September. For *Candidatus* genus *Rhizospheria*, Δ Ceq $_{rs, b}$ values were positive in all rhizospheres taken in July and September and in the leek rhizosphere in June. However, these were negative in the June samples from the grass and potato rhizospheres. Remarkably, *Candidatus* genus *Rhizospheria* was specifically enhanced in the rhizosphere of leek as compared to bulk soil and the other two rhizospheres.

The different acidobacterial subdivisions did not always prefer rhizosphere over bulk soils. In concrete terms, the Δ Ceq $_{rs, b}$ values of subdivision 1 acidobacteria were negative in all grass and potato rhizospheres, zero in the leek rhizosphere in June and July, indicating no effect of plant roots on subdivision 1 acidobacteria, and slightly positive in the leek rhizosphere in September (about two-fold higher in the leek rhizosphere than in bulk soil). Representatives of this subdivision thus grossly remained unaffected in the leek rhizosphere where they were stimulated in their growth later in the season. For subdivision 3 acidobacteria, Δ Ceq $_{rs, b}$ values were negative in all rhizospheres in June and in the grass and potato rhizospheres in July, were zero in the leek rhizosphere in July and in the grass and potato rhizospheres in September. Again these were positive in the leek rhizosphere in September (about four-fold higher than in bulk soil). For subdivision 4 acidobacteria, the Δ Ceq $_{rs, b}$ values were negative in the leek rhizosphere in June, positive in the grass and leek rhizospheres in September

Figure 3. Luteolibacter, *Candidatus* genus *Rhizospheria*, subdivisions 1, 3, 4, 6 acidobacteria and holophaga as percentage of total bacteria in grass, potato and leek rhizospheres and bulk soil. Bars marked with 'A' indicate significant higher fraction than in corresponding bulk soil.
Lut, *Luteolibacter*; Rhiz, *Candidatus* genus *Rhizospheria*; AS1, AS3, AS4, AS6, respectively, subdivision 1, 3, 4, 6 acidobacteria; Holo, holophaga. Bars on top of columns represent standard errors of means.
doi: 10.1371/journal.pone.0082443.g003

Figure 4. Biplot diagram calculated by redundancy analysis (RDA) on total bacteria, *Luteolibacter* and *Candidatus* genus *Rhizospheria* and of subdivisions 1, 3, 4 and 6 acidobacteria and holophaga as species, and location in soil, plant species, sampling time and soil pH as environmental variables. Environmental factors marked with * have significant effects on species variables at a significance level of P = 0.002.
doi: 10.1371/journal.pone.0082443.g004

(respectively two and eight-fold higher than in bulk soil) and close to zero in all other samples. Representatives of this subdivision thus remained grossly unaffected in the potato rhizosphere. For subdivision 6 acidobacteria, the Δ Ceq $_{rs,\ b}$ values were close to zero in June and July and positive in all three rhizospheres in September (between five and 25-fold higher than in bulk soil). Moreover, members of this subdivision had a stronger preference for grass and potato rhizospheres than for the one of leek. For Holophaga, the Δ Ceq $_{rs,\ b}$ values were positive in all leek rhizospheres across time (between four and 16-fold higher than in bulk soil), whereas they were negative or zero in the ones of grass and potato. The behavior of *Holophagae* in the three rhizospheres was thus different from that of all other subdivisions of the phylum *Acidobacteria*, in the sense that this group showed a strong preference for the leek rhizosphere throughout the experimental time period.

Contribution of *Bacteria, Acidobacteria* subdivision 1, 3, 4, 6, *Holophagae*, *Luteolibacter* and *Candidatus* genus *Rhizospheria* to total bacterial community in bulk and rhizosphere soils

Luteolibacter, Candidatus genus *Rhizospheria* and acidobacteria/ Holophaga numbers, expressed as percentage of total bacteria, were between 2.00 . 10^{-5} and . 91.6 over all groups (Figure 3). The relative abundances of subdivisions 1, 3, 4 and 6 acidobacteria in all three rhizospheres were equal to, or significantly lower, than those in corresponding bulk soils. For *Luteolibacter*, *Candidatus* genus *Rhizospheria* and Holophaga, the relative abundances in the rhizospheres of grass and potato were also equal to, or significantly lower than those in corresponding bulk soil, the exception being the grass rhizosphere in July, where the fraction of *Candidatus* genus *Rhizospheria* was significantly higher than in bulk soil. However, in all leek rhizospheres, the relative abundances of these three groups were always significantly higher than in bulk soil, with one exception (i.e. Holophaga in September, whose relative abundance was equal to the one in bulk soil). This indicates that *Luteolibacter*, *Candidatus* genus *Rhizospheria* and Holophaga are competitive towards other bacteria in the rhizosphere of growing leek plants where their abundances, relative to the total bacterial community, is higher than in bulk soil.

Factors affecting different bacterial populations in field soil

The effects of sample type, time and pH as environmental variables on cell estimates of all studied groups, as species variables, were calculated by multivariate analysis (RDA). A total of 93.3% of all variation was explained by the first two RDA axes (Figure 4). The rhizosphere of leek versus that of grass was discriminatory for most of the different studied populations. Subdivisions 1 and 3 acidobacteria and Holophaga correlated with the leek rhizosphere, whereas bacteria and subdivision 6 acidobacteria correlated with the grass rhizosphere.

The factors soil pH and 'grass rhizosphere' correlated with each other, indicating that either one or both are discriminative for bacteria and subdivision 6 acidobacteria. Subdivision 4 acidobacteria, *Luteolibacter* and *Candidatus* genus *Rhizospheria* did not show strong correlations with grass or leek rhizospheres.

Selection of *Holophagae* and of strain CHC25 in experimental leek-soil microcosms

A Kuchenbuch-style experimental plant-soil microcosms, the same used to assess the rhizosphere competence of *Luteolibacter* and *Candidatus* genus *Rhizospheria* strains [12], was applied to assess the competence of indigenous *Holophagae* (non-sterile soil) and of *Holophagae* strain CHC25 (following introduction into sterilized soil).

In non-sterilized non-inoculated soil, the average Holophaga cell number estimate (expressed as log Ceq per g dry soil) in the zone between 0 and 2 mm from the membrane that separated leek roots from the soil was 4.90 (range between 4.87 and 4.95). In a zone beneath, between 10 and 12 mm, the average log cell estimate was significantly (n=3, $P \leq 0.05$)

Figure 5. Colonization of the leek rhizosphere by *Holophagae* and strain CHC25 in soil. Holophaga cell equivalent numbers were compared in non-sterile soil and sterilized soil (set up A) or in sterilized soil inoculated with strain CHC25 (set up B) at 0-2 mm and 10-12 mm distances from the nylon gauze with roots (A), and between 0-2 mm layers of the systems where a 1-cm of sterilized non-inoculated soil layer was placed between sterilized soil inoculated with strain CHC25 and the nylon gauze with (set up C), or without leek roots (set up D) (B). Bars on top of columns represent standard errors of means. SED = Standard error of difference; * or **, significantly different at levels of, respectively, 0.01 ≤ P < 0.001 and P ≤ 0.001.
doi: 10.1371/journal.pone.0082443.g005

lower, i.e. 4.24 (4.19 - 4.29) (Figure 5). This indicates that *Holophagae* naturally present in the soil increase in number when proximate to leek roots, confirming the observations made in the field.

Upon introduction into sterilized soil without leek plants, the strain CHC25 cell numbers persisted between estimated average log values of 4.32 (after 1 d) and 5.14 (after 21 d) for the duration of the experiment (35 days). In sterilized soil with added strain CHC25 cells planted with leek, the average cell number estimate in the 0-2 mm zone was 5.90 (5.86 - 5.93), i.e. significantly higher than in the same layer of the system without growing leek plants (5.24, range between 5.03 - 5.27). This number was also significantly higher than in the 10-12 mm layers of both systems (5.14, range between 4.91 - 5.36). In the experiment in which a one-centimeter layer of sterilized non-inoculated soil was placed between the strain CHC25-inoculated soil and the membrane with leek roots, the estimated average log value in the 0 - 2 mm layer after 35 days was 5.24 (5.08 - 5.45) per g. In the same system without leek roots, this value was at background level, i.e. 3.52 (3.43 - 3.65) per g. This background estimate was statistically indistinguishable from the one measured in sterilized non-inoculated soil without plants (3.57, range between 3.39 - 3.75). These data strongly indicate that the strain CHC25 cells in soil are selected by leek roots at a distance of at least 10 mm in soil. Likely, cells migrated to direct influence of leek roots (rhizosphere/rhizoplane), where they occupied available sites and multiplied. We posit, therefore, that particular member of class *Holophagae*, as represented by strain CHC25, should be considered as rhizosphere-competent.

Discussion

A field experiment was designed to explore the responses of five subdivisions of *Acidobacteria* and of two distinct groups within *Verrucomicrobia* subdivision 1 to the roots of different plant species. The selected subdivisions were found to be erratically present in one or more of the rhizospheres studied. Strikingly, we obtained compelling evidence for the contention that members of the *Holophagae* are competent in the leek rhizosphere. Leek rhizosphere competence has been shown before for *Verrucomicrobia* subdivision 1, exemplified by *Candidatus* genus *Rhizospheria* [12]. Hence, particular *Acidobacteria* can be common in rhizospheres, which is consistent with earlier reports on the presence of members of this phylum in the rhizospheres of *Lolium perenne* and *Trifolium repens* [41], Lodgepool pine [9], different grasses (*Stipa hymenoides* and *Hilaria jamesii*) [42], taxus [43], *Thlaspi goesingense* [44], chestnut [6] and oilseed rape (*Brassica napus*) [45]. This also implies that the roles of *Acidobacteria* in the rhizosphere can be complex. Further work will need to address the precise roles of different *Acidobacteria* that are found to be competent in the rhizospheres of particular plant species.

Our data are relevant for the current understanding on the interactions of predominant soil bacteria with the roots of different plants, as hardly anything is known about the association of the often numerically dominant members of the *Acidobacteria* and *Verrucomicrobia* with these. Representatives of both groups are often difficult to culture and hence most ecological studies in plant-soil environments have been performed with molecular tools that target entire phyla, thereby ignoring the behavior of specific subgroups within such phyla. An important message from this study, and the ones of Nunes da Rocha et al. [12] and Navarrete et al [22], is that, given their widely divergent ecological behavior, more attention needs to be paid to the behavior of the individual groups within the *Acidobacteria* and *Verrucomicrobia*, e.g. via isolation and re-introduction strategies. The strains that are isolated can be used for studying interactions with plants under selected experimental conditions. Validation of our subdivision and subgroup-specific quantification systems by making use of cultivable representatives of the different groups, allowed us to proximate actual cell number in the studied soil compartments over time. In the assumption that maximally one to two 16S ribosomal gene copies will be present in the genomes of different *Verrucomicrobia* [46] and *Acidobacteria* groups [47,48], cell equivalent numbers may proximate actual cell numbers if the genome numbers per cell remain constant for the different groups in the different soil compartments. So far, it is unknown to which extent the genome copy number per cell of the typical rhizosphere-responsive groups, such as *Candidatus* genus *Rhizospheria* and *Holophagae*, increases in the neighborhood of plant roots. Eventual increases in genome copy numbers in these groups may lead to an over-estimation of cell numbers near leek roots. Other confounding factors like presence of plant-derived (chloroplast) DNA in rhizosphere extracts can be excluded to influence bacterial quantities in different rhizospheres. Namely, no plant-specific amplicon sequences were found upon PCR amplification of rhizosphere soil DNA with the same bacterial primers as was used in our study [49], and only a small fraction of amplicons of non-bacterial origin were found after bacterial PCR amplification and high throughput sequencing from rhizosphere soil

processed according the same procedure as applied in our study [50].

Remarkably, we found evidence supporting the fact that *Holophagae* as group, or a particular subset thereof, specifically responded to leek roots by an increase in 16S rRNA gene copy number and not to the ones of potato and grass growing in the same field. Moreover, removal of the grass layer covering the field resulted in an increase in the *Holophagae* 16S rRNA gene copy number later during the season in bulk soil, which allows the hypothesis that grass roots can be suppressive towards *Holophagae*. Grass and leek both are monocotyledonous plant species and hence the preference of *Holophagae* for cannot be explained along the monocot/dicot dichotomy. The lack of a stimulatory effect of the dicot potato in the field indicated that local conditions established by the roots were not propitious to holophagal cell growth. This in spite of the fact that pH in the potato rhizosphere was indistinguishable from that in the leek rhizosphere.

Our observations thus shed new light on the lifestyles of particular soil *Acidobacteria*. *Acidobacteria* commonly are considered to encompass mainly oligotrophic or K strategist forms [15,24,25,51]. The likely presence of low-specificity / high-affinity substrate uptake systems, as evidenced from analyses of the genomes of *Acidobacteria* subdivision1 and subdivision 3 strains [48], may indicate that these strains indeed exhibit oligotrophy as a major lifestyle in soil. This stands in sharp contrast to the here-defined *Holophagae* lifestyle, which was clearly responsive to leek roots, either by increased cell division and/ or by increase in genome quantity per cell, showing typical r-strategist behavior. As is the case for many other lineages within the bacterial domain [24], a clear niche differentiation exist among species of the phylum *Acidobacteria*.

The rhizosphere of leek thus appears to represent a specific niche for the *Holophagae* species that were studied. Two strains, CHC25 and ORAC (> 1300 bp stretches of the rRNA gene sequences were deposited in the EMBL Nucleotide Sequence Database, respectively, under accession numbers FN554392 and FN689719) , were able to grow on simple organic acids common in root exudates like oxalic acid, malic acid, succinic acid and citric acid [52]. Both strains closely resembled each other and substantially differed in taxonomy and physiology from *Holophagae* strains *Geothrix fermentans* H-5 [53] and *Holophaga foetida* TMBS4 [54]. Whereas the latter two strains are obligatory anaerobic, our strains were aerobic [52]. In their physiologies, strains CHC25 and ORAC resembled the aerobic *Holophagae* strain Acanthopleuribacter *pedis* FYK2218^T [55]. Cells of this strain are also motile as was the case for our strains CHC25 and ORAC [52]. However, the taxonomical distance between our strains and *A. pedis* strain FYK22218^T was larger than with *G. fermentans* H-5/ *H. foetida* TMBS4 [32]. Thus, strains CHC25 and ORAC potentially represent a new species, clearly distinct from any previously described *Holophagae* species. We tentatively propose the name for strains CHC25 and ORAC as *Candidatus* Porrumbacterium *oxyphilus*, a leek bacterium that prefers oxygen; the ecologically-defined traits that clearly distinguish strains CHC25 and ORAC from other strains within the class *Holophagae*.

In conclusion, we found specialized groups within several subdivisions of *Verrucomicrobia* and *Acidobacteria* that are rhizosphere competent. Their lifestyles with the plant may suggest that these bacteria either interact with the plants themselves or with communities associated with plants. This novel insight extends our current understanding of bacteria that associate with plants and may be a basis for further exploration of their putative roles in other habitats.

Acknowledgements

We thank Pieter Kastelein and Vladimir Fediy for their assistance in the plant-soil microcosm experiments.

Author Contributions

Conceived and designed the experiments: JDvE LSvO CMP UNdR. Performed the experiments: UNdR IG. Analyzed the data: LSvO UNdR IG CMP. Contributed reagents/materials/ analysis tools: IG. Wrote the manuscript: LSvO UNdR.

References

1. Felske A, De Vos WM, Akkermans ADL (2000) Spatial distribution of 16S rRNA levels from uncultured acidobacteria in soil. Lett Appl Microbiol 31: 118-122.
2. Ge Y, Zhang JB, Zhang L-M, Yang M, He J-Z (2008) Long-term fertilization regimes affect bacterial community structure and diversity of an agricultural soil in Northern China. J Soils Sed 8: 43-50. doi: 10.1065/jss2008.01.270.
3. Hugenholtz P, Goebel BM, Pace NR (1998) Impact of culture-independent studies on the emerging phylogenetic view of bacterial diversity. J Bacteriol 180: 4765-4774. PubMed: 9733676.
4. Janssen PH (2006) Identifying the dominant soil bacterial taxa in libraries of 16S rRNA and 16S rRNA genes. Appl Environ Microbiol 72: 1719–1728. doi:10.1128/AEM.72.3.1719-1728.2006. PubMed: 16517615.
5. Jones RT, Robeson MS, Lauber CL, Hamady M, Knight R et al. (2009) A comprehensive survey of soil acidobacterial diversity using pyrosequencing and clone library analyses. ISME J 3: 442-453. doi: 10.1038/ismej.2008.127. PubMed: 19129864.
6. Lee S-H, Ka J-O, Cho J-C (2008) Members of the phylum *Acidobacteria* are dominant and metabolically active in rhizosphere soil. FEMS Microbiol Lett 285: 263-269. doi:10.1111/j. 1574-6968.2008.01232.x. PubMed: 18557943.
7. Schlesner H, Jenkins C, Staley JT (2006) The Phylum *Verrucomicrobia*: A Phylogenetically heterogeneous bacterial group. Prokaryotes 7: 881-896.
8. Nunes da Rocha U, Van Overbeek LS, Van Elsas JD (2009) Exploration of hitherto-uncultured bacteria from the rhizosphere. FEMS Microbiol Ecol 69: 313-328. doi:10.1111/j.1574-6941.2009.00702.x. PubMed: 19508698.
9. Chow ML, Radomski CC, McDermott JM, Davies J, Axelrood PE (2002) Molecular characterization of bacterial diversity in Lodgepole pine (*Pinus contora*) rhizosphere soils from British Columbia forest soils differing in disturbance and geographic source. FEMS Microbiol Ecol 42: 347-357. doi:10.1111/j.1574-6941.2002.tb01024.x. PubMed: 19709294.
10. DeAngelis KM, Brodie EL, DeSantis TZ, Andersen GL, Lindow SE et al. (2009) Selective progressive response of soil microbial community to wild oat roots. ISME J 3: 168-178. doi:10.1038/ismej.2008.103. PubMed: 19005498.
11. Kielak A, Pijl AS, Van Veen JA, Kowalchuk GA (2008) Differences in vegetation composition and plant species identity lead to only minor

changes in soil-borne microbial communities in a former arable field. FEMS Microbiol Ecol 63: 372-382. doi:10.1111/j.1574-6941.2007.00428.x. PubMed: 18205817.
12. Nunes da Rocha U, Van Elsas JD, Van Overbeek LS (2011) *Verrucomicrobia* subdivision 1 strains display a difference in the colonization of the leek (*Allium porrum*) rhizosphere. FEMS Microbiol Ecol 78: 297-303. doi:10.1111/j.1574-6941.2011.01156.x. PubMed: 21692818.
13. Sanguin H, Remenant B, Dechesne A, Thioulouse J, Vogel TM et al. (2006) Potential of a 16S rRNA-based taxonomic micro array for analyzing the rhizopshere effects of maize on *Agrobacterium* spp. and bacterial communities. Appl Environ Microbiol 72: 4302-4312. doi:10.1128/AEM.02686-05. PubMed: 16751545.
14. Zul D, Denzel S, Kotz A, Overmann J (2007) Effects of plant biomass, plant diversity, and water content on bacterial communities in soil lysimeters: Implications for the determinants of bacterial diversity. Appl Environ Microbiol 73: 6916-6929. doi:10.1128/AEM.01533-07. PubMed: 17873072.
15. Fierer N, Bradford MA, Jackson RB (2007) Toward an ecological classification of soil bacteria. Ecology 88: 1354-1364. doi:10.1890/05-1839. PubMed: 17601128.
16. Eichorst SA, Kuske CR, Schmidt TM (2011) Influence of plant polymers on the distribution and cultivation of bacteria in the phylum *Acidobacteria*. Appl Environ Microbiol 77: 586-596. doi:10.1128/AEM.01080-10. PubMed: 21097594.
17. Lauber CL, Hamady M, Knight R, Fierer N (2009) Pyrosequencing-based assessment of soil pH as a predictor of soil bacterial community structure at the continental scale. Appl Environ Microbiol 75: 5111-5120. doi:10.1128/AEM.00335-09. PubMed: 19502440.
18. Pankratov TA, Serkebaeva YM, Kulichevskaya IS, Liesack W, Dedysh SN (2008) Substrate-induced growth and isolation of *Acidobacteria* from acidic *Sphagnum* peat. ISME J 2: 551-560. doi:10.1038/ismej.2008.7. PubMed: 18309356.
19. Rousk J, Bååth E, Brookes PC, Lauber CL, Lozupone C et al. (2010) Soil bacterial and fungal communities across a pH gradient in an arable soil. ISME J 4: 1340-1351. doi:10.1038/ismej.2010.58. PubMed: 20445636.
20. Sait M, Davis KE, Janssen PH (2006) Effect of pH on isolation and distribution of members of subdivision 1 of the phylum *Acidobacteria* occurring in soil. Appl Environ Microbiol 72: 1852–1857. doi:10.1128/AEM.72.3.1852-1857.2006. PubMed: 16517631.
21. Liles MR, Turkmen O, Manske BF, Zhang M, Rouillard J-M et al. (2010) A phylogenetic microarray targeting 16S rRNA genes from the bacterial division *Acidobacteria* reveals a lineage-specific distribution in a clay fraction. Soil Biol Biochem 42: 739-747. doi:10.1016/j.soilbio.2010.01.007.
22. Navarrete AA, Kuramae EE, de Hollander M, Pijl AS, Van Veen JA et al. (2013) Acidobacterial community responses to agricultural management of soybean in Amazon forest soils. FEMS Microbiol Ecol 83: 607-621. doi:10.1111/1574-6941.12018. PubMed: 23013447.
23. Figuerola ELM, Guerrero LD, Rosa SM, Simonetti L, Duval ME, Galantini JA et al. (2012) Bacterial indicator of agricultural management for soil under no-till production. PLOS ONE7: e51075. doi:10.1371/journal.pone.0051075. PubMed: 23226466.
24. Phillippot L, Andersson SGE, Battin TJ, Prosser JI, Schimel JP et al. (2010) The ecological coherence of high bacterial taxonomical ranks. Nature Rev 8: 523-529. doi:10.1038/nrmicro2367.
25. Smit E, Leeflang P, Gommans S, van den Broek J, van Mil S et al. (2001) Diversity and seasonal fluctuations of the dominant members of the bacterial soil community in a wheat field as determined by cultivation and molecular methods. Appl Environ Microbiol 67: 2284–2291. doi:10.1128/AEM.67.5.2284-2291.2001. PubMed: 11319113.
26. Buckley DH, Schmidt TM (2001) Environmental factors influencing the distribution of rRNA from *Verrucomicrobia* in soil. FEMS Microbiol Ecol 35: 105-112. doi:10.1111/j.1574-6941.2001.tb00793.x. PubMed: 11248395.
27. Bruce T, Martinez IB, Maia Neto O, Vicente ACP, Kruger RH et al. (2010) Bacterial community diversity in the Brazilian Atlantic forest soils. Microb Ecol 60: 840-849. doi:10.1007/s00248-010-9750-2. PubMed: 20886336.
28. Bergmann GT, Bates ST, Eilers KG, Lauber CL, Caporaso JG et al. (2011) The under-recognized dominance of *Verrucomicrobia* in soil bacterial communities. Soil Biol Biochem 43: 1450-1455. doi:10.1016/j.soilbio.2011.03.012. PubMed: 22267877.
29. George IF, Hartmann M, Liles MR, Agathos SN (2011) Recovery of as-yet-uncultured *Acidobacteria* on dilute solid media. Appl Environ Microbiol 77: 8184-8188. doi:10.1128/AEM.05956-11. PubMed: 21948845.
30. Janssen PH, Yates PS, Grinton BE, Taylor PM, Sait M (2002) Improved culturability of soil bacteria and isolation in pure culture of novel members of the divisions *Acidobacteria, Actinobacteria, Proteobacteria*, and *Verrucomicrobia*. Appl Environ Microbiol 68: 2391-2396. doi:10.1128/AEM.68.5.2391-2396.2002. PubMed: 11976113.
31. Joseph SJ, Hugenholtz P, Sangwan P, Osborne CA, Janssen PH (2003) Laboratory cultivation of widespread and previously uncultured soil bacteria. Appl Environ Microbiol 69: 7210-7215. doi:10.1128/AEM.69.12.7210-7215.2003. PubMed: 14660368.
32. Nunes da Rocha U, Van Elsas JD, Van Overbeek LS (2010) Real-time PCR detection of *Holophagae* (*Acidobacteria*) and *Verrucomicrobia* subdivision 1 groups in bulk and leek (*Allium porrum*) rhizosphere soils. J Microbiol Methods 83: 141-148. doi:10.1016/j.mimet.2010.08.003.
33. Sangwan P, Kovac S, Davis KER, Sait M, Janssen PH (2005) Detection and cultivation of soil *Verrucomicrobia*. Appl Environ Microbiol 71: 8402-8410. doi:10.1128/AEM.71.12.8402-8410.2005. PubMed: 16332828.
34. Nunes da Rocha U, Andreote FD, De Azevedo JL, Van Elsas JD, Van Overbeek LS (2010) Cultivation of hitherto-uncultured bacteria belonging to the *Verrucomicrobia* subdivision 1 from the potato (*Solanum tuberosum* L.) rhizosphere. J Soils Sed 10: 326-339.
35. Lugtenberg B, Kamilova F (2009) Plant-growth-promoting rhizobacteria. Annu Rev Microbiol 63: 541-556. doi:10.1146/annurev.micro.62.081307.162918. PubMed: 19575558.
36. Houba VJG, Novozamsky I, Van Dijk D (1998) Certification of an air-dry soil for pH and extractable nutrients using one hundredth molar calcium chloride. Commun Soil Sci Plant Anal 29: 1083-1090. doi:10.1080/00103629809370010.
37. Lane DJ (1991) 16S/ 23S rRNA sequencing. In E StackebrandtM Goodfellow, Nucleic acid techniques in bacterial systematics. John Wiley and Sons, Chichester, UK. pp. 115-175.
38. Muyzer G, De Waal EC, Uitterlinden AG (1993) Profiling of complex microbial populations by denaturing gradient gel electrophoresis analysis of polymerase chain reaction-amplified genes coding for 16S rRNA. Appl Environ Microbiol 59: 695-700. PubMed: 7683183.
39. Pruesse E, Quast C, Knittel K, Fuchs BM, Ludwig W et al. (2007) SILVA: a comprehensive online resource for quality checked and aligned ribosomal RNA sequence data compatible with ARB. Nucleic Acids Res 35: 7188-7196. doi:10.1093/nar/gkm864. PubMed: 17947321.
40. Kumar S, Nei M, Dudley J, Tamura K (2008) MEGA; A biologist-centric software for evolutionary analysis of DNA and protein sequences. Brief Bioinform 9: 299-306. doi:10.1093/bib/bbn017. PubMed: 18417537.
41. Marilley L, Aragno M (1999) Phylogenetic diversity of bacterial communities differing in degree of proximity of *Lollium perenne* and *Trifolium repens* roots. Appl Soil Ecol 13: 127-136. doi:10.1016/S0929-1393(99)00028-1.
42. Kuske CR, Ticknor LO, Miller ME, Dunbar JM, Davis JA et al. (2002) Comparison of soil bacterial communities in rhizospheres of three plant species and the interspaces in an arid grassland. Appl Environ Microbiol 68: 1854-1863. doi:10.1128/AEM.68.4.1854-1863.2002. PubMed: 11916705.
43. Hao DC, Ge GB, Yang L (2008) Bacterial diversity of *Taxus* rhizosphere: culture-independent and culture-dependent approaches. FEMS Microbiol Lett 284: 204-212. doi:10.1111/j.1574-6968.2008.01201.x. PubMed: 18576948.
44. Idris R, Trifonova R, Puschenreiter M, Wenzel WW, Sessitsch A (2004) Bacterial communities associated with flowering plants of the Ni hyperaccumulator *Thlaspi goesingense*. Appl Environ Microbiol 70: 2667-2677. doi:10.1128/AEM.70.5.2667-2677.2004. PubMed: 15128517.
45. Macrae A, Rimmer DL, O'Donnell AG (2000) Novel bacterial diversity recovered from the rhizosphere of oilseed rape (*Brassica napus*) determined by the analysis of 16S ribosomal DNA. Antonie Van Leeuwenhoek 78: 13-21. doi:10.1023/A:1002745312030. PubMed: 11016691.
46. Wertz JT, Eunji K, Breznak JA, Schmidt TM, Rodrigues LM (2012) Genomic and physiological characterization of the *Verrucomicrobia* isolate *Diplosphaera colitermitum* gen. nov., sp. nov., reveals microaerophily and nitrogen fixation genes. Appl Environ Microbiol 78: 1544-1555. doi:10.1128/AEM.06466-11. PubMed: 22194293.
47. Eichorst SA, Breznak JA, Schmidt TM (2007) Isolation and characterization of soil bacteria that define *Terriglobus* gen. nov., in the phylum *Acidobacteria*. Appl Environ Microbiol 73: 2708–2717. doi:10.1128/AEM.02140-06. PubMed: 17293520.
48. Ward NL, Challacombe JF, Janssen PH, Henrissat B, Coutinho PM et al. (2009) Three genomes from the phylum *Acidobacteria* provide insight into the lifestyles of these microorganisms in soils. Appl Environ

Microbiol 75: 2046-2056. doi:10.1128/AEM.02294-08. PubMed: 19201974.
49. Fierer N, Jackson JA, Vilgalys R, Jackson RB (2005) Assessment of soil microbial community structure by use of taxon-specific quantitative PCR assays. Appl Environ Microbiol 71: 4117-4120. doi:10.1128/AEM.71.7.4117-4120.2005. PubMed: 16000830.
50. Inceoğlu Ö, Al-Soud WA, Salles JF, Semenov AV, van Elsas JD (2011) Comparative analysis of bacterial communities in a potato field as determined by pyrosequencing. PLOS ONE 6: e23321. doi:10.1371/journal.pone.0023321. PubMed: 21886785.
51. Wagner M, Horn M (2006) The *Planctomycetes*, *Verrucomicrobia*, *Chlamydiae* and sister phyla comprise a superphylum with biotechnological and medical relevance. Curr Opin Biotechnol 17: 241-249. doi:10.1016/j.copbio.2006.05.005. PubMed: 16704931.
52. Van Overbeek LS (2013) Exploration of Hitherto-Uncultured Bacteria from the Rhizosphere (chapter 16). In de Bruijn FJ, editor, Molecular microbial ecology of the rhizosphere. John Wiley and Sons, Chichester, UK.
53. Coates JD, Ellis DJ, Gaw VV, Lovley DR (1999) *Geothrix fermentas* gen. nov., sp. nov., a novel Fe(III)-reducing bacterium from a hydrocarbon-contaminated aquifer. Int J Syst Bacteriol 49: 1615-1622. doi:10.1099/00207713-49-4-1615. PubMed: 10555343.
54. Liesack W, Bak F, Kreft JU, Stackebrandt E (1994) *Holophaga foetida* gen. nov., sp. nov., a new homoacetogenic bacterium degrading methoxylated aromatic compounds. Arch Microbiol 162: 85-90. doi:10.1007/s002030050106. PubMed: 8085918.
55. Fukunaga Y, Kurahashi M, Yanagi K, Yokota A, Harayama S (2008) *Acanthopleuribacter pedis* gen. nov., sp. nov., a marine bacterium isolated from a chiton, and description of *Acanthopleuribacteraceae* fam. nov., *Acanthopleuribacterales*, ord. nov., *Holophagaceae* fam. nov., *Holophagales* or. nov. and Holophagae classis nov. in the phylum 'Acidobacteria'. Int J Syst Evol Microbiol 58: 2597-2601.
